# Serum 25-Hydroxyvitamin D Concentrations and Depressive Symptoms among Young Adult Men and Women

**DOI:** 10.3390/nu6114720

**Published:** 2014-10-28

**Authors:** Maria A. Polak, Lisa A. Houghton, Anthony I. Reeder, Michelle J. Harper, Tamlin S. Conner

**Affiliations:** 1 Department of Psychology, University of Otago, Dunedin 9054, New Zealand; E-Mail: tconner@psy.otago.ac.nz; 2 Department of Human Nutrition, University of Otago, Dunedin 9054, New Zealand; E-Mails: lisa.houghton@otago.ac.nz (L.A.H.); michelle.harper@otago.ac.nz (M.J.H.); 3 Cancer Society Social and Behavioural Research Unit, Department of Preventive and Social Medicine, University of Otago, Dunedin 9054, New Zealand; E-Mail: tony.reeder@otago.ac.nz

**Keywords:** vitamin D, depression, young adult, daily diary studies, vitamin D deficiency

## Abstract

There has been an increased interest in the role of vitamin D in depression; however, there have been few studies conducted in younger population groups. Our aim was to investigate the association between vitamin D status and depressive symptoms in a non-clinical young adult sample living in Dunedin, New Zealand. A cross-sectional sample of 615 young adults completed a questionnaire including demographics and the Centre for Epidemiological Studies Depression Scale (CES-D). Height, weight and a blood sample for 25-hydroxyvitamin D [25(OH)D] was obtained. Serum 25(OH)D was used to predict depression scores, adjusting for potential confounders including time spent outdoors for 13 consecutive days, BMI, age, sex and ethnicity. Prevalence of low vitamin D was high even in this age group, and serum 25(OH)D was negatively associated with depression symptoms before and after adjustment. When investigating the relationship between the presence* versus* absence of depressive symptoms and quartiles of 25(OH)D, participants in the lowest quartile were more likely to report depressive symptoms compared with those in the highest quartile. Although our findings suggest that vitamin D is a predictor of depression symptomatology, even when controlling for time spent outdoors, a randomised controlled trial in this young adult target group is needed to confirm the association.

## 1. Introduction

Recently, there has been an increased interest in the potential role of vitamin D in mental health and wellbeing. Vitamin D receptors are present in many different types of cells, including neurons and glial cells [1,2,3]. Most importantly, vitamin D is now accepted for its neurosteroid activity and impact on brain serotonin [2,4,5], suggesting a possible role in mood regulation. There is some evidence that low circulating concentration of serum 25-hydroxyvitamin D [25(OH)D], the marker for vitamin D status, is associated with mood disorders, including major depressive disorder, seasonal affective disorder, and premenstrual dysphoric disorder [6,7].

Sun exposure is the major source of vitamin D for humans [8,9], and thus, in the absence of adequate supplementation or suitable fortification, the concentration of circulating serum 25(OH)D decreases significantly during winter in non-equatorial locations [10]. At the same time, modern lifestyles often reduce the time in sunlight, decreasing opportunities to endogenously produce vitamin D in sufficient quantities [11]. Primary vitamin D deficiency is highly prevalent worldwide [12,13,14]. In European countries, 2% to 30% of all adults are estimated to be deficient in vitamin D, with up to 75% when considering older adults only [15]. A similarly high prevalence of vitamin D deficiency is present in most other areas, including those close to the equator [13].

While there is ample evidence of an association between serum 25(OH)D and mood disorders such as depression in older and vulnerable populations [6,16], there has been very little research on younger and non-clinical populations. Some studies in depressed populations did show evidence of less time spent outdoors in depressed individuals [17], leading to suggestion that depressive symptoms might keep people inside and thus limit their opportunities for the manufacture of endogenous vitamin D. Therefore, the aim of the present study was to investigate the association between vitamin D status and depression symptoms in a non-clinical sample of young men and women, taking into account time spent outdoors during daylight hours. We hypothesised that higher vitamin D status would be associated with lower depression scores from the CES-D controlling for gender, age, ethnicity, BMI, and the time spent outdoors during daylight hours.

## 2. Experimental Section

### 2.1. Participants

Participants were healthy university student volunteers recruited through the University of Otago, which is located in Dunedin, New Zealand (45°52′0″S). Participants were recruited for the Daily Life Study, a cross-sectional study of experiences and biological markers of wellbeing carried out between March and May (southern hemisphere autumn) during the academic years 2011 and 2012. Inclusion criteria were as follows: aged between 17 and 25 years, enrolled at the University of Otago, and access to the Internet. Of the total 666 participants who completed the initial survey, 615 participants completed at least 7 of 13 daily diaries and provided a venous blood sample. The study was approved by the University of Otago Human Ethics Committee (#10/177). All participants provided written informed consent.

### 2.2. Procedure

Participants who fulfilled the inclusion criteria were invited to an onsite session where they completed an initial computerised survey including the demographics variables and depression scale and obtained instructions for completing the 13-day online diaries (day 1 of study). Starting on day 2 of the study, participants completed an online daily diary for 13 consecutive days including a question about time spent outdoors that day. The diary took about 5–10 min to complete and also included questions about other daily experiences of participants (not part of the current study). The diary was available from any on- or off-campus computer through a password-protected website between 3 p.m. and 8 p.m. After completing the 13-day daily diary protocol, participants attended a session in the Human Nutrition Clinic of the University of Otago between 8.30 a.m. and 11.00 a.m. (day 15 of the study). During the clinic visit, their height and body composition were measured using standardised techniques, and they provided a non-fasting venous blood sample for 25(OH)D analysis.

### 2.3. Measures

Participants’ depressive symptoms were assessed using the Center for Epidemiologic Studies Depression Scale (CES-D). The CES-D has been developed specifically for research in the general population (*i.e.*, not clinical evaluation). The scale consists of 20 items that encompass four areas: depressed affect, positive affect, somatic vegetative signs, and interpersonal distress, with an emphasis on the affective component [18]. Participants rated how often they experienced symptoms associated with depression, including problems sleeping, poor appetite, and feeling sad over the past week. Response options range from 0 to 3 for each item (0 = rarely or none of the time, 1 = some or little of the time, 2 = moderately or much of the time, 3 = most or almost all the time). Four items (4, 8, 12 and 16) are worded positively and were reverse coded for analyses. Six participants did not answer one CES-D item each, and individual means were imputed. The final score was obtained by calculating the total sum across all items, which could range from 0 to 60, with higher scores indicating greater presence of depressive symptoms. A total score of 16 or higher is equivalent to experiencing five or more symptoms all or most of the time and is indicative of possible clinical depression [18].

To measure time spent outdoors, we modified the sun habits questionnaire developed by Glanz and colleagues [19] to create one question suitable for close-to-real-time reporting of actual daily sun exposure, asking participants to report on the time they spent outdoors during daylight hours on that day. This question was presented in the daily diary. Participants selected one of 12 categories ranging from 0 to 15 min to 10 h or more, and the answers were scored from 0 to 11.

During the clinic visit (day 15), participants’ height and weight were measured using standardised techniques [20]. All measurements were taken in duplicate by the same trained anthropometrist using calibrated equipment (Seca) with the participants wearing light clothing and no shoes. The mean of the two closest measurements was calculated. A third measure was taken if the first two measures were outside the acceptable range of variance for the given measurement. These measures were used to calculate body mass index [BMI (kg/m^2^)]. Participants also provided a venous non-fasting blood sample for serum total 25(OH)D analyses.

### 2.4. Blood Sample Processing and 25(OH)D Analysis

Following blood collection, the samples were centrifuged at 3000 rpm for 15 min at 4 °C, the serum was aliquoted then stored at −80 °C until analysis. Serum 25(OH)D was analysed using isotope-dilution liquid chromatography tandem mass spectrometry based on the method of Maunsell* et al.* [21], using an API 3200 instrument (Applied Biosystems) connected to a Dionex Ultimate 3000 HPLC system. The limit of quantification for the assay was <5 nmol/L. To assess accuracy and inter-assay variability, external quality control serum material (UTAK Laboratories) containing low and medium concentration of 25(OH)D_3_ and 25(OH)D_2_ were analysed with every run.

A different batch of controls was used for each year of the study. In 2011, the 25(OH)D_3_ low control mean was 26.0 nmol/L (verified value 27.5 nmol/L); CV 3.8%, and the medium control mean 75.7 nmol/L (verified value 77.5 nmol/L); CV 2.9%. For 25(OH)D_2_, the low control mean was 22.5 nmol/L (verified value 24.2 nmol/L); CV 7.0% and the medium control mean 71.2 nmol/L (verified value 72.7 nmol/L); CV 4.5%.

In 2012, the 25(OH)D_3_ low control mean was 29.6 nmol/L (verified value 29.9 nmol/L); CV 3.1%, and the medium control mean 86.1 nmol/L (verified value 79.9 nmol/L); CV 3.7%. For 25(OH)D_2_ the low control mean was 24.8 nmol/L (verified value 26.6 nmol/L); CV 6.2% and the medium control mean 79.5 nmol/L (verified value 77.5 nmol/L); CV 4.9%.

Internal quality control pooled serum samples were also analysed; the inter-assay CV for 25(OH)D_3_ was 5.8% at 20.3 nmol/L (2011) and 3.9% at 60.7 nmol/L (2012). The concentration of 25(OH)D_2_ in the internal controls was below the limit of quantification for both years.

### 2.5. Statistical Analyses

Descriptive statistics were used to summarise the demographics and anthropometric characteristics of our sample including age, gender, and BMI. Descriptive variables were reported as means (standard deviation) by gender for all participants. We calculated a mean score for time spent outdoors during daylight hours for each participant by averaging each person’s scores across the maximum 13 daily diaries. Mean values were used instead of the total score since we did not have the same number of daily reports for each participant. We performed a linear regression analysis to examine the relationship between vitamin D status as a continuous predictor variable and depression as a continuous outcome variable taking into account gender, age, ethnic background, and BMI. We controlled for gender (coded 0 for men and 1 for women) because women showed both higher depressive symptoms and higher 25(OH)D concentrations than men in our sample. We controlled for age, even though we had a narrow age range, for two reasons: the prevalence of depression changes with age [22], and the efficiency of manufacturing vitamin D in response to sunlight can also vary with age [3]. Ethnicity categories were NZ European, Māori/Pacific Islander, Asian and Other in line with the top level ethnicity classification used by Statistics New Zealand. Dummy variables were created for the Māori/Pacific Islander, Asian, and Other categories with NZ European as the reference category. Finally, to test whether time outdoors explains the association between 25(OH)D and depression, we added average time spent outdoors score as a control variable. Statistical analyses were carried out using the IBM SPSS Statistics package version 19 (IBM Corp. Released 2010. IBM SPSS Statistics for Windows, Armonk, NY, USA.).

## 3. Results

The mean age of participants was 19.5 years (*SD* = 1.5; range 17 to 25 years), 75% were in their first or second year of university, and more than half were female (62%; 381/615). The majority of participants were of European origin (79.8%), 9.9% were Asian, 2.9% Māori or Pacific Islander, and 7.4% of other ethnicities. Of our sample, 2.3% (14/615) were classified as underweight (BMI < 18.5), 68.5% as normal weight (BMI 18.5–24.9), while 23.3% and 6.0% of our sample were classified as overweight (BMI 25–29.9) and obese (BMI ≥ 30), respectively. Sample characteristics did not differ statistically across the two years of data collection. There were also no statistically significant differences in total serum 25(OH)D and total CES-D scores between 2011 and 2012.

CES-D, serum 25(OH)D concentrations and confounding variables are reported in Table 1. The mean total serum 25(OH)D concentration was 64.1 nmol/L (SD = 26.6 nmol/L) ranging from 8.2 nmol/L to 177.0 nmol/L. When participants were divided into groups by gender, differences were found in CES-D scores, serum 25-hydroxyvitamin D, and BMI (Table 1). Total CES-D score in our sample ranged from 0 to 46 with 219 participants (35.4%) scoring 16 or above, representing the presence of depressive symptoms indicative of clinical depression. The mean score for time spent outdoors was 2.0 (SD = 1.2), meaning that, on average, participants spent between half an hour to one hour outside per day. Average time spent outdoors during daylight hours was not a significant predictor of depressive symptoms or 25(OH)D. Participants with a CES-D score of 16 or higher had lower 25(OH)D even though they did not differ on mean time spent outdoors (Table 2).

**Table 1:** Average CES-D score, serum 25(OH) vitamin D, time spent outdoors, and BMI for all participants, and men and women separately.

Variable	ALL (*n* = 615)		Men (*n* = 234)		Women (*n* = 381)		
	Mean	(SD)	Mean	(SD)	Mean	(SD)	
CES-D score	14.1	(8.4)	12.9	(8.2)	14.8	(8.5)	*p* = 0.005
Serum 25(OH) D (nmol/L)	64.1	(26.6)	56.5	(21.9)	68.7	(28.2)	*p* < 0.001
Time spent outdoors score	2.0	(1.2)	2.2	(1.4)	1.8	(1.1)	*p* = 0.001
BMI	23.6	(3.7)	24.1	(3.7)	23.4	(3.7)	*p* = 0.014

Abbreviations: CES-D, Center for Epidemiologic Studies Depression Scale; BMI, body mass index; *p* value is based on the results of a *t*-test comparing men and women.

**Figure 1:**
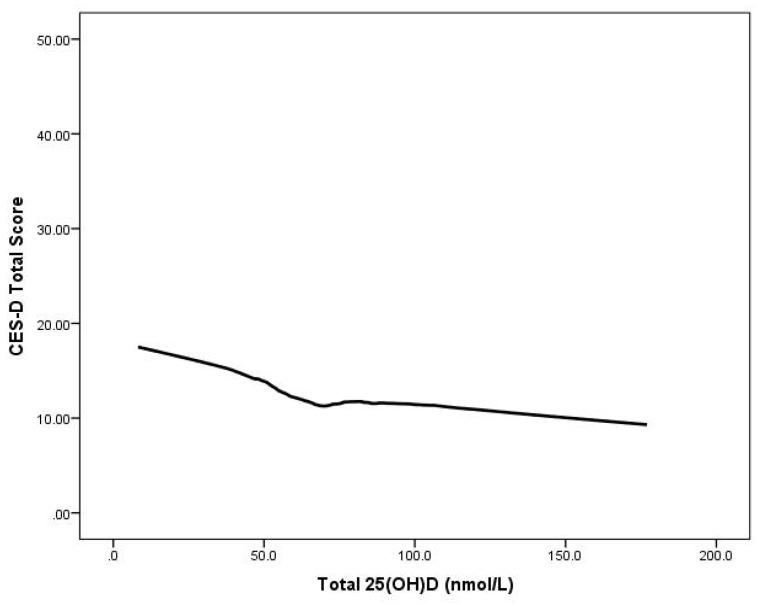
CES-D scores by serum 25(OH)D concentration in 615 young adults aged 17–25 years: LOWESS plot.

Multiple regression analyses showed that serum 25(OH)D status was significantly negatively associated with the total CES-D scores (controlling for gender, age, BMI, and ethnicity) (B = −0.048, *t* = −3.828, *p* < 0.001) Moreover, the relationship between 25(OH)D and depression scores remained significant even after adjusting for mean time outdoors (B = −0.053, *t* = −3.567, *p* < 0.001. (Figure 1). Additionally, we examined the relationship between the presence* versus* absence of depressive symptoms and quartiles of 25(OH)D adjusted for age, gender, ethnic origin, BMI and time spent outdoors (Table 3). Participants in quartiles I to III and compared with those in the highest quartile were more likely to report depressive symptoms; however, this relationship was present for men but not women.

**Table 2:** Participant characteristics by presence of depressive symptomatology (CES-D score below 16* vs.* 16 and over).

Variable	ALL (*n* = 615)		CES-D score < 16 (*n* = 396)		CES-D score ≥ 16 (*n* = 219)		
	Mean	(SD)	Mean	(SD)	Mean	(SD)	
CES-D score	14.1	(8.4)	8.9	(3.7)	23.4	(6.3)	*p* < 0.001
Serum 25(OH) D (nmol/L)	64.1	(26.6)	67.4	(26.3)	58.0	(26.2)	*p* < 0.001
Time spent outdoors score	2.0	(1.2)	2.0	(1.1)	2.0	(1.4)	*p* = 0.783
BMI	23.6	(3.7)	23.8	(3.9)	23.4	(3.4)	*p* = 0.220

Abbreviations: CES-D, Center for Epidemiologic Studies Depression Scale; BMI, body mass index; *p* value is based on the results of a *t*-test comparing participants with CES-D scores <16 and ≥16.

**Table 3:** Adjusted odds ratios (95% confidence intervals) for the presence of depressive symptoms (CES-D ≥ 16) and 25-hydroxyvitamin D quartile.

25(OH)D Quartile (Range in nmol/L)	*n*	All	*n*	Men	*n*	Women
IV (≥80.0)	155	reference	27	reference	128	reference
III (64.0–79.9)	153	1.34 (0.81, 2.23)	62	2.51 (1.14, 5.50)	91	0.64 (0.31, 1.32)
II (44.0–63.9)	156	2.22 (1.27, 3.86)	75	3.21 (1.34, 7.63)	81	1.51 (0.72, 3.18)
I (<5.0–43.9)	151	2.24 (1.27, 3.97)	70	7.14 (1.83, 27.78)	81	1.96 (0.62, 2.59)

Adjusted for age, gender, age, ethnic origin, BMI, and time spent outdoors.

## 4. Discussion

In this population-based study of over 600 young adults, lower vitamin D status was associated with higher depression scores even after taking potential confounders including time spent outdoors into consideration. While several studies have evaluated the relationship between vitamin D and depression among older adults [23,24], the relationship in younger adults has not yet been investigated. On average, in practical terms, our analysis showed that for every one standard deviation increase in serum 25(OH)D (27 nmol/L), there was a 4.5 point decrease in the CES-D score among our participants aged 17–25 years.

To date, few large epidemiological studies have been performed in a population-based setting; however, our results are in accordance with survey-based findings in the USA and Europe. Specifically, Ghanji and colleagues utilizing data reported in the U.S. National Health and Nutrition Examination Survey (NHANES) found an independent association between lower 25(OH)D concentration and higher depression status and depression severity among a group of participants aged 15–39 years (*n* = 7970) surveyed between 1988 and 1994 [25]. In their study, depression status was assessed using the Diagnostic Interview Schedule (DIS) developed by the National Institute of Mental Health. In a large sample of older adults aged 65–95 years randomly selected from 11 municipalities in the Netherlands, Hoogendijk and colleagues also demonstrated an association of depression status and depression severity assessed using CES-D with decreased serum 25(OH)D concentration [24]. Lee and colleagues also reported an association between 25(OH)D concentration and depression assessed using BDI-II among a large sample of middle-aged and older European men [26]. Another case control study found low concentration of 25(OH)D associated with depressive disorder in 18–65-year-old participants in the Netherlands [27].

In contrast to our findings, Zhao and colleagues found no significant association between serum 25(OH)D concentrations and the presence of depression among 3916 U.S adults aged 20 years and over from the 2006-06 NHANES [28]. Participants’ depressive symptoms were assessed using the Patient Health Questionnaire (PHQ-9) which is a standard instrument for use in depression diagnosis in primary care [29]. Pan and colleagues also reported no significant association between vitamin D status and depressive symptoms in 3262 Chinese older adults aged 50–70 years [30].

There are a number of possible reasons for the conflicting results among studies, including differences in population groups (*i.e.*, ethnic and other cultural differences), age of the participants surveyed, measures of depression obtained, type of assay used for the analysis of serum vitamin D, and statistical adjustment of known confounding factors including sun exposure. Importantly, in a recent study by Nanri and colleagues, the authors found an association between 25(OH)D and depression symptoms during winter, but not in summer or overall [31], thereby suggesting the season of study has a major influence on results.

One of the strengths of our study is that vitamin D status was measured using the nominal gold standard method. Other strengths include the inclusion of several variables that may influence depression outcome in the data analysis—in particular, taking into account time spent outside as a proxy for recent sun exposure. This is in contrast to the recent findings that higher levels of reported sun exposure, rather than 25(OH)D concentration, were associated with less depressive symptoms in sample of multiple sclerosis patients [32]. However, the authors used a different instrument—a recall questionnaire rather than a close-to-real time diary of the current study and they appropriately highlight the need to consider reverse causality as an explanation for the findings between sun exposure and depression. Interestingly, we found no association between average daily time spent outdoors during a recent period relevant for subcutaneous vitamin D manufacture and 25(OH)D concentration in our study participants. Even though we obtained daily reports of time spent outdoors for each day on 13 consecutive days, this measure only serves as a proxy of potential opportunities to produce endogenous vitamin D, and vitamin D status is ultimately affected by exposure longer than two weeks. Overall sun exposure and thus the production of vitamin D varies in individuals not only as a function of time spent outdoors, but also time of day, clothing, and current weather conditions [33]. In addition, we did not have information about the exact time of day participants spent the time outside, actual weather, and sunscreen use by participants during the time they were outside during the two-week study period. Moreover, a plateau of vitamin D production is reached after about 15–30 min of sun exposure [9], suggesting that additional time outdoors may not provide additional benefits as far as vitamin D production is concerned. Future studies could consider using real-time reporting of time outdoors including current weather conditions and actual use of sun protection. However, such close monitoring of sun exposure and sun protective behaviours could lead to behavioural changes during the study due to social expectations of sun protection use.

Limitations of the study included the cross-sectional design, which limits the ability to evaluate the cause and effect relation between depression and vitamin D deficiency; however, from a public health perspective, the coexistence of vitamin D deficiency and depression in this sample of young adults is a concern. Depression and chronic low mood is a major global health problem with concomitant consequences on morbidity, mortality, and quality of life. Although the prevalence of major depression is relatively low among community-dwelling adults, the occurrence of subsyndromal depression or depressive symptomatology is high and estimated to affect around 12% of adults [34,35]. Our sample mean for the CES-D score of 14.1 was very similar to that found in a previous study of self-reported mood scales in the general population [22], suggesting that we have obtained a representative sample of young adults on the dimension of depression. Interestingly, over one-third of our participants had a total CES-D score of 16 or above, indicating the possibility of the presence of clinical depression. Most importantly, a recent study elaborated on the possible mechanism of vitamin D effects on the serotonin system implicated in depression [36]. The presence of vitamin D is likely to upregulate transcription of serotonin-synthesising gene tryptophan hydroxylase 2 (TPH2) in the brain and concurrently to downregulate the transcription of THP1 outside the brain [5].

## 5. Conclusions

The present study found evidence of an association between 25(OH)D, a marker of vitamin D status, and depression scores in a young adult sample. The association of vitamin D and depression remained significant even after adjusting for age, gender, ethnicity, BMI, and time spent outdoors. Our results support the previous findings, suggesting a direct effect of vitamin D on the serotonin system that should be followed up with an appropriately designed, randomised controlled trial of supplementation with vitamin D among young adults in the general population.
